# PREVALENCE AND CORRELATES OF MILD COGNITIVE IMPAIRMENT AND DEMENTIA IN OLDER KOREAN AMERICANS

**DOI:** 10.1093/geroni/igz038.1514

**Published:** 2019-11-08

**Authors:** Hochang Lee, Miyong Kim, Peter Walsh, Kim B Kim, Haera Han

**Affiliations:** 1 University of Rochester Medical Center, Rochester, New York, United States; 2 University of Texas at Austin, Austin, Texas, United States; 3 University of Rochester, Rochester, New York, United States; 4 Korean Resource Center, Ellicott City, Maryland, United States; 5 Johns Hopkins University, Baltimore, Maryland, United States

## Abstract

With a high prevalence of well-established risk dementia risk factors (e.g. tobacco, alcohol use, hypertension, and diabetes), there is a dire need for data on prevalence and correlates of cognitive impairment and related service utilization among older Korean Americans. Memory and Aging study of Koreans (MASK) is a two-stage epidemiological survey with two-step sampling strategy to obtain a representative sample. We screened 1,118 older Korean Americans (mean age: 70.7, female: 67.2%) with a Korean version of the Mini-Mental State Exam, and 118 persons sampled from two levels of performance on the MMSE underwent the second stage clinical evaluation that included the Consortium to Establish a Registry for Alzheimer’s Disease (CERAD). Preliminary analysis estimated that community prevalence of mild cognitive impairment in this population was 28.4% (CI: 18.2%-41.5%) and dementia was 7.2% (3.8% - 13.2%). Correlates of cognitive impairment, and potential barriers to the services will be discussed during this presentation.

